# Long-term prediction of Iranian blood product supply using LSTM: a 5-year forecast

**DOI:** 10.1186/s12911-024-02614-z

**Published:** 2024-07-29

**Authors:** Ebrahim Miri-Moghaddam, Saeede Khosravi Bizhaem, Zohre Moezzifar, Fatemeh Salmani

**Affiliations:** 1 Blood Transfusion Research Center, High Institute for Research and Education in Transfusion Medicine, Tehran, Iran; 2 Department of Molecular Medicine, Faculty of Medicine and Cardiovascular Diseases Research Center, Birjand University of Medical Sciences, Birjand, Iran; 3 Cardiovascular Diseases Research Center, Birjand University of Medical Sciences, Birjand, Iran; 4 Department of Epidemiology and Biostatistics, School of Health, Cardiovascular Diseases Research Center, Birjand University of Medical Sciences, Birjand, Iran

**Keywords:** Blood products, LSTM, ARIMA, Deep learning

## Abstract

**Background:**

This study aims to predict the trend of procurement and storage of various blood products, as well as planning and monitoring the consumption of blood products in different centers across Iran based on artificial intelligence until the year 2027.

**Methods:**

This research constitutes a time-series investigation within the realm of longitudinal studies. In this study, information on the number of packed red blood cells (RBC), leukoreduced red blood cells (LR-RBC), and platelets (PLT), PLT-Apheresis, and fresh frozen plasma (FFP) was requested from all blood transfusion centers in the country and extracted using a unified protocol. After the initial examination of the information and addressing data issues and inconsistencies, the corrected data were analyzed. Both conventional and artificial intelligence approaches were used to predict each product in this study. The best model was selected based on goodness-of-fit indicators RMSE and MAPE.

**Results:**

Based on the obtained results, the FFP product will follow a relatively consistent process similar to previous years in the next five years. The PLT product is predicted to have a growing trend over the next 5 years, which applies to both the demand and supply of the product. The PLT-Apheresis product also shows a similar upward trend, albeit with a lower growth rate. The RBC product will have a constant trend over a 5-year period (long-term) according to both models, taking into account short-term changes. Similarly, there is a similar trend in LR-RBC, with the expectation that short-term pattern repetition will continue over a 5-year period (long-term). Comparing the goodness-of-fit results, the LSTM model proved to be better for predicting the dominant blood products.

**Conclusions:**

The growth of the elderly population and diseases related to old age, and on the other hand, the trend of increasing the consumption of the product with a short lifespan (PLT) requires the activation of the management of the patient’s blood, especially in relation to this product in medical centers. The trend for other products in the next five years is similar to previous years, and no growth in demand is observed. The LSTM method, considering periodic and cyclical events, has performed the prediction.

## Introduction

Blood is a valuable and limited resource with no satisfactory alternative, necessitating the optimal use of blood products, especially in light of the global aging population [1]. The challenge of providing sufficient and healthy blood, especially during crises, requires appropriate policies tailored to each community’s needs and resources [2]. Despite advancements making blood transfusion safer, it still carries significant side effects, akin to other therapeutic interventions [3]. Excessive requests for blood products in teaching hospitals create issues such as improper distribution, escalating costs, increased workload for blood banks, and higher expenses and complications associated with blood transfusion [4]. Improper blood use incurs substantial costs for healthcare systems, including collection, testing, storage, and management expenses [4]. Blood transfusion plays a vital role in the recovery and control of patients undergoing surgery [5]. Over time, it has become an integral part of disease treatment protocols. According to 2019 data from the World Health Organization (WHO), 117.4 million blood donations were made globally, with 42% in high-income countries representing 16% of the world’s population. In low-income countries, approximately 52% of blood transfusions are given to children under five, while in high-income countries, 75% are administered to patients over 65. Disparities in plasma-derived medicinal product (PDMP) production exist, with only 50 out of 173 countries producing them, 83 countries importing all PDMPs, 24 not using them, and 16 not responding to the question [6]. The unpredictable balance between blood supply and demand, influenced by changing lifestyles and eligibility criteria, has intensified due to population growth and age-related diseases. Understanding the consumption rate of diverse blood products is crucial for predicting essential facilities and equipment needs, facilitating resource allocation planning, and managing blood bag inventory. Additionally, it aids in comprehending clinical demand and ensuring necessary screening facilities in blood banks. Predicting the required amount of blood products for each country is crucial for effective crisis management.

Iran is one of the member countries of WHO’s Global Database on Blood Safety and is located in the Eastern Mediterranean region. The blood needed was provided in 91 centers between 2013 and 2018. In Iran, 65% of donations were given by donors aged 25–44. Donors aged 45 to 64 years and 18–24 years contributed 25% and 7% of the donations, respectively, and 3% were given by donations younger than 18 years. Blood donated by first-time donors decreased from 432,687 to 245,412 between 2014 and 2018 2018, but the amount of donations did not decrease in Iran. Blood donated through apheresis procedures has increased 13 times between these years. The number of positive tests for blood-transmitted infection markers decreases from 68 to 51 cases in the mentioned years. The most changes in blood product consumption were reported in plt-apheresis between 2014 and 2018 [7].

Prediction is usually done using various methods, one of which is the use of time series-based approaches. Unlike regression modeling, time series add complexity and sequential dependency among input variables. Utilizing deep learning methods alongside time series can provide more accurate predictions. One powerful type of neural network designed for handling sequential dependencies is recurrent neural networks (RNNs). Long short-term memory (LSTM) networks are a type of RNN used in deep learning. The LSTM method is a variation of an RNN proposed by Hochreiter and Schmidhuber in 1997. Typically, RNNs have short-term memory as they utilize past information for the current neural network. Conventional recurrent neural networks can lose information, often referred to as the “vanishing gradient” problem, which arises from the repeated use of recurrent weight matrices. Therefore, in this study, we aim to perform more accurate predictions for the management of blood and blood product requirements in various national centers, including procurement, preparation, evaluation, storage, and consumption planning, by employing LSTM network.

## Materials and preliminaries

### Data collection and missing data management

Data was collected retrospectively from the national blood transfusion database of the entire country according to a standardized protocol. The protocol and data extraction checklist were prepared and approved based on the guidelines of the National Blood Transfusion Organization. Subsequently, the approved protocol was sent to all 38 blood transfusion centers in the country, and data for the past 10 years was extracted on a monthly basis. The outputs were collected in a consistent format. Then, after aggregating the data and removing any abnormal or erroneous entries through examination and validation, the analysis was performed on the remaining data. Due to the absence of recorded data for certain months in some centers, we relied on information from time points with consecutive records. In instances where data was missing within sequence, we utilized a neighboring imputation method to estimate and fill in the missing values.

### Time series models

Time-series is referred to as an ordered sequence of events related to a special variable at equal time intervals. Time-series analysis has been applied for many purposes, such as economic forecasting, utility studies, stock market analysis, medical sciences, and epidemic forecasting. There are many statistical forecasting procedures for time-series model. Auto-regressive (AR) [8] and moving average (MA) [9] models are two main time-series models mentioned in the literature. Furthermore, combination of these two models, such as ARMA and ARIMA, has been widely used for forecasting time-series trends [10]. Box-Jenkins Analysis is a systematic approach for identifying, fitting, checking, and utilizing integrated autoregressive, moving average (ARIMA) time series models. This method is particularly suitable for analyzing time series data of medium to long length [11]. Two fuzzy approaches have also been proposed for forecasting, namely ANFIS [12] and fuzzy-time series models [13].

### Long short-term memory (LSTM) model

LSTM, which was proposed by Hochreiter and Schmidhuber in 1997, is a specific kind of RNN that tackles the issue of vanishing gradients and effectively handles long-term dependencies in sequential data. Unlike conventional RNNs, LSTM integrates memory cells and gates that allow for selective retention or dismissal of information across multiple time steps [14].

In an LSTM model, the recurrent weight matrix is replaced with a specific function in the cycle and controlled by a set of gates. The input gate, output gate, and forget gate act as switches that control the weights and create the functionality of long-term memory. With recurrent cycles, gradients can become either very large or very small, and with each iteration, the combination of network gradients in each direction becomes easier. This combination can make the gradients either too large or too small. While gradient explosion is a weakness of traditional RNNs, LSTM architecture significantly mitigates these issues.

After making a prediction, the prediction is fed back into the model to forecast the next value in the sequence. With each prediction, an error is introduced to the model. To prevent gradient explosion, values are passed through sigmoid and tanh functions before being used as input and output. By using these gates, LSTM can learn to retain important information for long periods and discard irrelevant or redundant information. This capability makes LSTM particularly effective in modeling and predicting complex sequential patterns [14–17].

In this study, 70% of the dataset was allocated for the training phase, while the remaining portion was set aside for testing and validation purposes. In the train section, a closed-loop forecasting approach was employed. Within this phase, the LSTM neural network is trained to forecast the succeeding time step’s value at each step of the input sequence. Furthermore, to enhance compatibility and mitigate overfitting during the training process, predictors and objectives underwent normalization to achieve a mean of zero and a variance of one. In the prediction phase, the test data were likewise normalized using a comparable method employed for the training data. To define the LSTM, a layer with 130 hidden units was utilized. The number of units was not increased further to prevent overfitting.

### Goodness of fit indexes

There are various methods available for measuring the accuracy of prediction models. The most common one is the Root Mean Squared Error (RMSE), which calculates the square root of the average of prediction errors. Additionally, metrics such as Mean Absolute Percentage Error (MAPE), is also used. In these metrics, a lower value indicates a better fit of the model. The calculation method for each metric will be explained further.

$$\begin{gathered} RMSE=\sqrt {\frac{1}{n}\sum\limits_{{t=1}}^{n} {e_{t}^{2}} } \hfill \\ MAPE=\frac{{100\% }}{n}\sum\limits_{{t=1}}^{n} {\left| {\frac{{{e_t}}}{{{y_t}}}} \right|} \hfill \\ \end{gathered}$$

In these indexes, the symbol “e” represents the difference between the observed and predicted values, and “y” represents the observed values at time “t”.

## Results

Both the deep learning and classical models have predicted an upward trend in the distribution levels of RBC products. According to the observations, the product has increased from 1,407,075 in 2011 to 1,478,566 in 2016. Extending this upward trend, the deep learning method predicts it to reach 1,485,297.92 in 2027, starting from 1,293,029.49 in 2022. On the other hand, the classical method forecasts the product to reach 1,443,865.24 in 2027, starting from 1,415,716.90 in 2022 (Table 1).

**Table 1:** Forecasted number of blood products for five years in Iran using LSTM and classical methods

Product	Method	2011	2016	2021	2022	2023	2024	2025	2026	2027
RBC	LSTM	1,407,075	1,478,566	1,296,856	1,293,029	1,267,109	1,420,861	1,567,420	1,508,190	1,485,298
	Classic	1,407,075	1,478,566	1,296,856	1,415,717	1,421,347	1,426,976	1,432,606	1,438,236	1,443,865
	CI95%	1,224,253	1,330,552	1,100,356	1,145,210	933937.2	719811.6	491842.8	248194.6	196194.9
		1,568,592	1,649,650	1,419,077	1,686,224	1,908,756	2,134,141	2,373,368	2,628,277	2,898,951
RBC LR	LSTM	259786.1	409488.6	456,731	478929.8	429013.5	409537.7	453578.1	480291.2	420467.1
	Classic	259786.1	409488.6	456,731	504225.5	526206.9	536380.3	541088.7	543267.9	544276.5
	CI95%	205008.8	350605.7	390009.4	433,137	401867.2	361371.8	320999.7	283337.7	248788.3
		305313.1	450909.9	490313.7	575,314	650546.7	711388.8	761177.8	803198.1	839764.6
PLT	LSTM	703122.7	892037.3	855,511	881806.8	903386.7	896568.4	937048.2	879951.4	903732.5
	Classic	703122.7	892037.3	855,511	944102.1	968636.5	993170.8	1,017,705	1,042,239	1,066,774
	CI95%	603311.7	799373.3	732966.6	799764.9	719118.7	633055.3	538453.1	434974.2	322829.1
		808630.5	990893.1	924332.1	1,088,439	1,218,154	1,353,286	1,496,957	1,649,505	1,810,719
FFP	LSTM	630,345	708176.3	609,615	668516.1	708262.1	730768.5	704688.2	677149.7	636995.4
	Classic	630,345	708176.3	609,615	615414.8	611380.3	611021.7	608541.3	607264.4	605304.9
	CI95%	591955.5	667201.1	575843.6	561875.9	551763.5	538778.7	529527.8	520,064	511684.1
		682,038	755598.7	664241.2	668953.7	670997.2	683264.7	687554.9	694464.8	698925.7
PLT-Apheresis	LSTM	2582.86	10435.71	30,305	29022.83	29054.02	29528.37	30136.8	30740.65	30913.43
	Classic	2582.86	10435.71	30,305	32059.96	34806.7	37576.86	40,347	43117.15	45887.3
	CI95%		5561.7	26782.94	24908.91	21876.87	18857.83	15507.34	11766.2	7626.94
			13938.08	35104.19	39211.01	47736.53	56295.87	65186.66	74468.11	84147.67

In the case of the LR-RBC product, the reported results were 259,786.08 in 2011 and 409,488.61 in 2016. The deep learning method predicts a level of 478,929.76 in 2022, while the classical method estimates it to be 504,225.53. In the year 2027, the LSTM method predicts a level of 420,467.09, whereas the classical method forecasts it to be 544,276.46 (Table 1).

For the PLT- Apheresis product, the results show an increase from 2,582.86 in 2011 to 29,022.83 in 2022 and 30,913.43 in 2027, indicating a consistent trend. However, a rising trend is predicted from 32,059.96 in 2022 to 45,887.30 in 2027. Based on goodness-of-fit indices, the classical method shows higher accuracy in this product (Table 1).

The results of the PLT product were reported as 703,122.67 in 2011 and 892,037.33 in 2016. For the prediction in 2023, the LSTM method estimates 881,806.82, while the classical method provides an estimate of 944,102.11. In 2027, the deep learning method predicts 903,732.45, and the classical method predicts 1,066,773.83 (Table 1).

In the case of the FFP product, the results show an increase from 630,345 in 2011 to 708,176.32 in 2016. The LSTM method predicts 668,516.11, and the classical method predicts 615,414.80 for the year 2022. In 2027, the LSTM method predicts 636,995.43, while the classical method predicts 605,304.88 (Table 1).

The distribution chart of the LR-RBC product is presented. As evident in both charts, the classical method has overestimated compared to the LSTM method. The LSTM method has provided predictions by identifying long-term trends and short-term fluctuations, including downward points. An important point to note is the absence of overshooting in the LSTM method compared to the classical method. The monthly observed and predicted distribution chart of the PLT- Apheresis product is provided. In both requested and distributed products, an upward trend is observed in the product’s forecast.

**Fig. 1:**
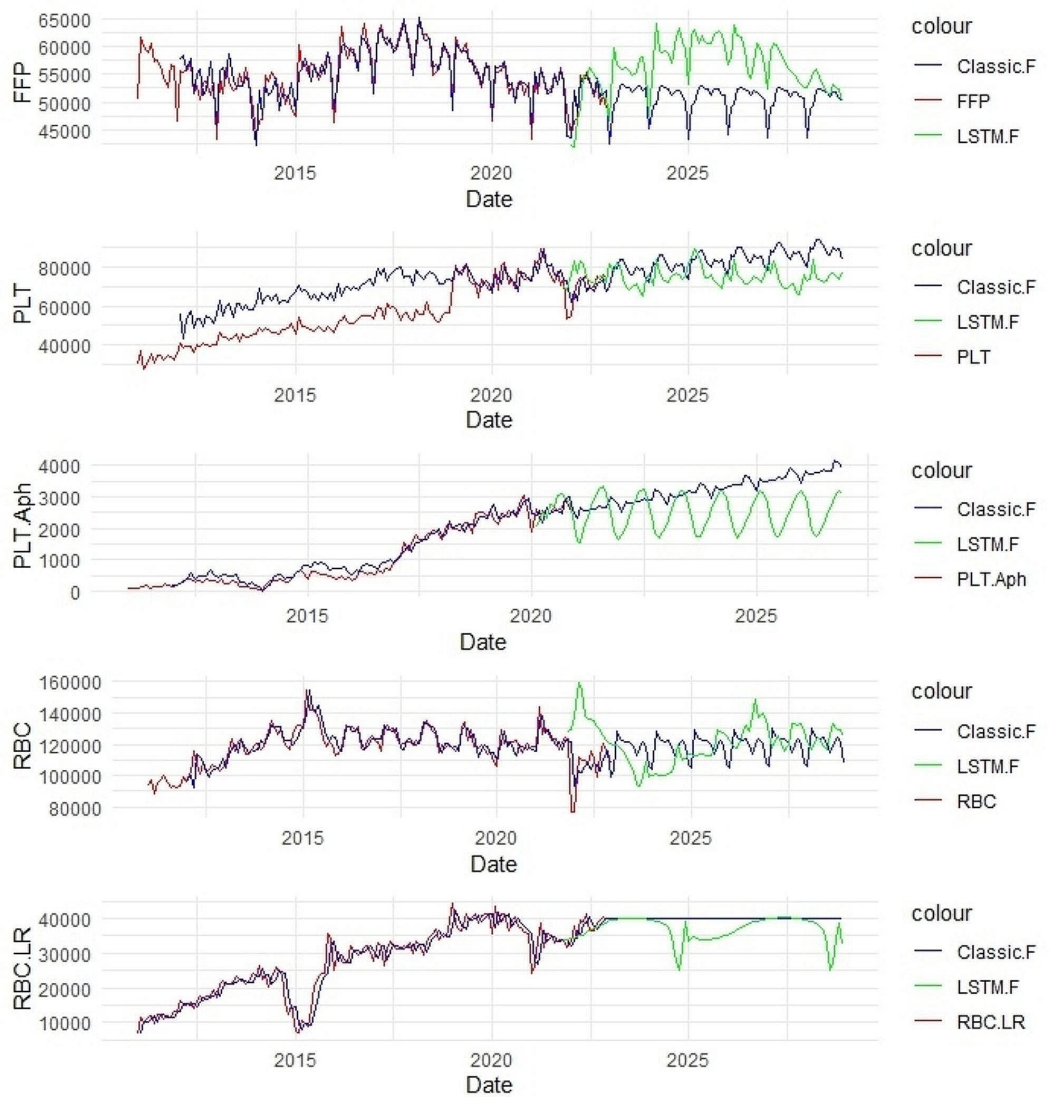
Prediction of blood product requirements until 2027 using classical and LSTM methods

The process of the required blood products for shipment over consecutive years, along with a 5-year forecast using both mentioned methods, is depicted in Fig. 1.

This study utilized two approaches, classical (MA, AR, ARMA, ARIMA, SARIMA) and deep learning, for forecasting the products. The deep learning approach was implemented based on the Long Short-Term Memory (LSTM) neural network model. A comparison between the two approaches was conducted using two evaluation metrics, RMSE and MAPE. Since a lower goodness-of-fit criterion indicates a better model, the RMSE and MAPE values were calculated for each product (RBC, LR-RBC, FFP, PLT, PLT- Apheresis) using both classical and LSTM methods, for the FFP, RBC, and LR-RBC products, the LSTM method has lower MAPE values compared to the classical method, indicating that it is more suitable for forecasting these products. For the PLT product, both methods exhibit similar accuracy, while the classical method provides higher accuracy for the PLT- Apheresis product (Table 2).

**Table 2:** Goodness of fit index

	FFP		RBC		RBCLR		PLT		PLT-Apheresis	
	Classic	LSTM	Classic	LSTM	Classic	LSTM	Classic	LSTM	Classic	LSTM
RMSE	1998.20	1984.01	1835.20	1921.21	2419.64	2332.64	6993.82	6112.50	210.56	271.14
MAPE	0.03	0.05	0.05	0.05	0.05	0.05	0.08	0.10	0.07	0.09

## Discussion

Demand and supply management of blood and its derivatives are considered a significant challenge in the healthcare system. Obtaining healthy blood involves careful donor selection, adherence to guidelines, and rigorous quality control measures. The global process of blood preparation and delivery to healthcare centers is costly, irrespective of voluntary donations.

The study utilized ARIMA, exponential smoothing, and LSTM deep learning methods to analyze trends and forecast blood product consumption in Iran, comparing their goodness of fit. Similar approaches, ARIMA, and exponential smoothing, were employed by Volken et al. for forecasting RBC demand in Switzerland [18]. Guo et al. employed autoregressive methods to forecast pediatric red blood cell demand. They introduced SARIMA as a highly accurate short-term prediction model, effectively simulating monthly usage trends in a time series [19]. In predicting various blood products across different centers, the SARIMA model emerged as the top-recommended choice, aligning with the study’s findings. The adoption of deep learning methods, particularly LSTMs, has recently garnered attention for their capacity to learn long-term dependencies in sequential data. Notably, Shung et al.‘s study on predicting red blood cell transfers in acute gastrointestinal bleeding patients showcased the LSTM method’s superior accuracy compared to other regression-based approaches, affirming parallels with our study [20]. Qi et al. addressed blood shortages between stations and institutions by enhancing the LSTM method with the GOV approach, predicting RBC levels effectively [21]. Schilling et al. highlighted platelet product management challenges and utilized both LASSO and LSTM methods, finding similar accuracy in predicting platelet demand. This aligns with our study, reinforcing LSTM as a suitable method for blood product prediction [22].

For the RBC product, a consistent 5-year trend is expected, with deep learning showing a growing trend in short-term demand, contrasting with the classical method indicating a constant trend. In the case of LR-RBC, a consistent trend aligning with previous years is expected, in line with the classical method, but the deep learning method estimates a lower demand due to long-term changes. This trend is consistent with the demand rate.

In 2019, the United States saw a 5.1% decrease in the collection of red blood cell units compared to 2017, with 11,590,000 units collected. However, the transfusion of red blood cell units increased by 2.5% to 10,852,000 units [23]. Globally, changes in red blood cell utilization were noted among countries reporting to the European Blood Alliance. Between 2016 and 2017, most countries reported a 2.2% overall decrease, but between 2017 and 2018, 7 out of 18 countries showed an increase, while 11 reported a 1.1% overall decrease [24–26]. Various regions, including New Zealand, Australia, Quebec, and England, report changes in RBC demand, with many countries exhibiting lower usage rates per population than the United States (33.1 units of RBC per 1000 people). In 2019, RBC injection rates per 1000 people were 20.9 in New Zealand, 24.2 in Quebec, 24.7 in Australia, and 24.6 in England [24–26]. The reasons for these differences are unclear, suggesting potential opportunities for the U.S. to further reduce RBC use. In 2018, Anam University Hospital implemented a modified Patient Blood Management (PBM) program. Over the study period, the hospital observed a decrease in RBC transfusions per 10,000 patients from 139.8 in 2018 to 137.3 in 2019. The proportion of patients receiving RBC transfusions with hemoglobin levels below 7 g/dL increased, indicating more appropriate blood transfusion practices. The implementation of the PBM program and ongoing education for healthcare personnel played significant roles in these positive outcomes.

This study predicts a growing trend in demand and distribution of apheresis platelets, despite limited data. Platelets derived from whole blood, analyzed through classical and deep learning methods, show a consistent trend with deep learning estimating lower values in 2026 and 2027. Apheresis platelets are preferred for chronic platelet consumers like leukemia patients due to their quality and better performance, leading to increased usage. In the United States, between 2017 and 2019, platelet distribution decreased by 2.0%, but platelet transfusion increased by 15.8%, possibly driven by the rising incidence of cancer and chronic diseases among the elderly. The study highlights the significance of apheresis platelets and the evolving landscape of platelet transfusion practices [23, 27, 28]. Between 2017 and 2019 in the United States, PLT transfusion increased across various healthcare settings, reflecting a rising demand driven by clinical conditions in the elderly population [23]. In an Austrian hospital study, the average use of platelet concentrates slightly decreased after implementing PBM. Platelet concentrate variations ranged from 76 to 198 units between 2013 and 2017 [29]. Anticipating increased demand for apheresis platelets in Iran, challenges in procurement and supply are foreseen, compounded by an aging donor population. Apheresis platelet production, requiring more time than whole blood donation, poses difficulties in attracting and retaining younger donors. Platelets’ shorter shelf life compared to red blood cells and plasma further complicates supply. Potential strategies to address these challenges include optimizing donor recruitment, adopting technologies for enhanced platelet storage, and utilizing platelet units from whole blood.

The study predicts a continued availability of FFP over the next five years, maintaining a relatively consistent process despite the need for significant decision-making in response to a growing trend. In the United States between 2017 and 2019, plasma distribution and transfusion both declined by 16.5% and 8.0%, respectively [23]. In Austria, a twelve-year study revealed a substantial decrease in plasma unit use (26.1% decrease) after implementing PBM. The findings underscore the evolving landscape of plasma utilization, with a need for informed decision-making in response to changing trends [29].

Despite improved blood consumption management, the future is marked by an anticipated increase in the demand for blood products, primarily due to aging populations. A potential shortage could lead to an estimated 5.6 million patient deaths in the United States alone. Globally, someone requires a life-sustaining blood transfusion every second [30]. Guidelines like MSBOS offer comprehensive frameworks for optimal blood consumption in healthcare centers, emphasizing the importance of maintaining a proper cross-match-to-transfused ratio [31]. Unsafe transfusion practices and unnecessary injections pose risks of adverse reactions and transfusion-transmissible infections for patients [32]. The WHO recommends the “Safe Blood” strategy for universal access to secure blood and improved safety. However, challenges such as reduced blood bank reserves, higher procurement costs, improper distribution, and increased workload for transfusion services persist. Many hospitals face excess demand for blood, causing significant financial and moral losses. Regular evaluation of blood consumption rates is crucial for transfusion medicine and laboratory specialists, aiding in supply and demand management and identifying reasons for trend change [33]. PBM is a key strategy for reducing allogeneic blood transfusion, focusing on evidence-based approaches to understand blood demand and consumption [34]. The WHO advocates for the establishment of systems, such as hospital blood transfusion committees and blood care, to monitor and enhance the safety of blood transfusion processes. Currently, 128 countries have national guidelines on the clinical use of blood. Blood transfusion committees are found in 48% of hospitals conducting transfusions, with varying prevalence across income levels: 62% in high-income countries and 35% in upper-middle-income countries [30]. Recent healthcare advancements emphasize strengthening supply chains, particularly in the blood supply chain spanning from donors to patients. Patient blood needs drive this chain, with a focus on reducing waste, shortages, overall healthcare costs, and improving public health, patient safety, and service quality. Inaccurate information on demand volume can lead to ordering decisions causing resource waste and increased healthcare costs, especially concerning products with short shelf lives. Overstocking blood products, beyond necessary levels, not only incurs substantial costs for transfusion centers but also compromises blood unit quality, contributing to increased waste.

In this study, our limitation was associated with the monthly submission of aggregated data on blood product derivatives. Due to the monthly consolidation of the number of sent derivatives as a input variable, we were unable to conduct an analysis of individual demographic characteristics for both donors and recipients on a person-to-person basis.

In summary, the available information suggests a growing demand for PLT and PLT-Apheresis products, while other products show a trend similar to previous years with no observed growth in demand over the next five years. The growth of the elderly population and diseases related to old age, and on the other hand, the trend of increasing the consumption of the product with a short lifespan (PLT) requires the activation of the management of the patient’s blood, especially in relation to this product in medical centers. It’s important to note that predictive studies are subject to conditions existing in historical data. The study utilized the LSTM method, accounting for periodic and cyclical events, for its predictions.

## Data Availability

The datasets used and analyzed during the current study are available from the corresponding author on reasonable request.
